# Prevalence and Risk Factors of self-reported dry eye in Brazil using a short symptom questionnaire

**DOI:** 10.1038/s41598-018-20273-9

**Published:** 2018-02-01

**Authors:** Julia Silvestre de Castro, Iara Borin Selegatto, Rosane Silvestre de Castro, Eliana C. M. Miranda, José Paulo Cabral de Vasconcelos, Keila Monteiro de Carvalho, Carlos Eduardo Leite Arieta, Monica Alves

**Affiliations:** 1 Faculty of Medical Sciences, University of Campinas – UNICAMP, São Paulo, Brazil; 2 Faculty of Medical Sciences of Santos, UNILUS, São Paulo, Brazil

## Abstract

To evaluate dry eye prevalence and investigate associated risk factors in Brazil by applying a short questionnaire of symptoms and risk factors. A cross-sectional study of 3,107 participants from all the five different geopolitical regions of Brazil. Overall prevalence of dry eye in this study population was 12.8%. Dry eye previous diagnosis was reported by 10.2% and presence of severe symptoms in 4.9%. Logistic regression analysis confirmed some significantly risk factors, such as female sex (Odds Ratio (OR) 1.74; 95% Confidence Interval (CI): 1.12–1.93), age ≥60 year-old (OR 2.00; 95%CI: 1.44–2.77), history of ocular surgery (OR 1.84; 95%CI: 1.30–2.60), contact lens wear (OR 1.93; 95%CI: 1.36–2.73), cancer treatment (OR 3.03; 95%CI: 1.36–6.59), computer use >6 hours per day (OR 1.77; 95%CI: 1.36–2.31), antidepressants (OR 1.61; 95%CI: 1.12–2.31) and anti-allergy (OR 2.11; 95%CI: 1.54–2.89) medications. Nevertheless, when stratified by regions, each one had its own significant factors and inherent characteristics. This is the first study about prevalence and risk factors of dry eye in a large population sample from all regions of Brazil. Dry eye is a common condition in the Brazilian population and prevalence rates varies substantially in the different geographic regions of the country, possibly reflecting climate and socioeconomic discrepancies.

## Introduction

Dry eye is a common, complex and multifactorial disease of the ocular surface and tear film that results in discomfort and visual disturbance^1–3^. In recent years, several basic science and clinical studies have increased our knowledge about dry eye mechanisms and associated risk factors^4,5^. In 2007 the report of the epidemiology subcommittee of the Dry Eye Workshop (DEWS) was published, summarizing the available evidence about prevalence, risk factors and impact of dry eye^6^. One of the issues raised in this first report was the relative lack of population-based studies of dry eye, which included mostly studies from North America, Australia and Asia (China and Indonesia)^7–13^. Although considerable progress has been achieved in the description of dry eye epidemiology since this report, it should be noted that these new studies comprise data from Europe and other Asian countries, as well as updated information about previously described populations^14–24^, with large areas of the globe such as Africa, Middle East and Latin America remaining with no data about dry eye prevalence, as recently pointed by the DEWS II epidemiology report^25^.

Brazil is the largest country in South America, covering an area of 8 516 000 km², larger than continental USA. According to the last census released in 2010, Brazil had a population of 190 million inhabitants^26^ but already reached to 208 million in 2017 following population actual projections made by the official agency of statistics^27^. Historically, Brazilian population has been made up of a confluence of people mixed with native indigenous groups with Portuguese colonizers, black African slaves and lately European, Asian and Arabic emigrates. So, it is, characterized by deep miscegenation and is asymmetrically distributed among the main 5 geopolitical regions of the country which present diverse climate particularities ranging from humid equatorial to semi-arid areas, which may potentially influence diseases prevalence and risk factors impacts.

As dry eye is considered a symptomatic condition, accurate evaluation of symptoms is of paramount importance for its diagnosis in the context of epidemiological studies. Accordingly, several epidemiological studies defined dry eye as the presence of severe symptoms such as dryness and irritation based on the application of short questionnaires, such as Women Health Study^9,17,23,28–30^. The short dry eye questionnaire used herein has been previously applied in other studies, with a high specificity for dry eye^31^. Indeed, it was recently translated into Portuguese and validated in the pilot study made by our group^32^. Therefore, this study aims to evaluate dry eye prevalence and investigate associated risk factors in Brazil by applying a short questionnaire of symptoms and risk factors.

Prevalence and risk factors of dry eye was evaluated in a population sample of 3,107 individuals from 5 different geopolitical regions of Brazil, providing the largest and most comprehensive report about the prevalence of dry eye in Latin America.

## Results

The present study enrolled 3,107 participants from the five geopolitical regions of Brazil, with a mean age of 40.5 (±17.1) years old and female: male ratio of 1.9:1.0. 4,000 questionnaires were distributed by regular mail, from capitals to small size cities and 3,107 of which returned, yielding a 77.7% rate of participation. This questionnaire comprises 3 items about previous diagnosis of dry eye and a range of dryness and irritation symptoms and has been extensively used in population-based studies worldwide. It was previously translated and validated, to than be applied as a cross-sectional survey in all five different geopolitical regions of Brazil.

**Figure 1:**
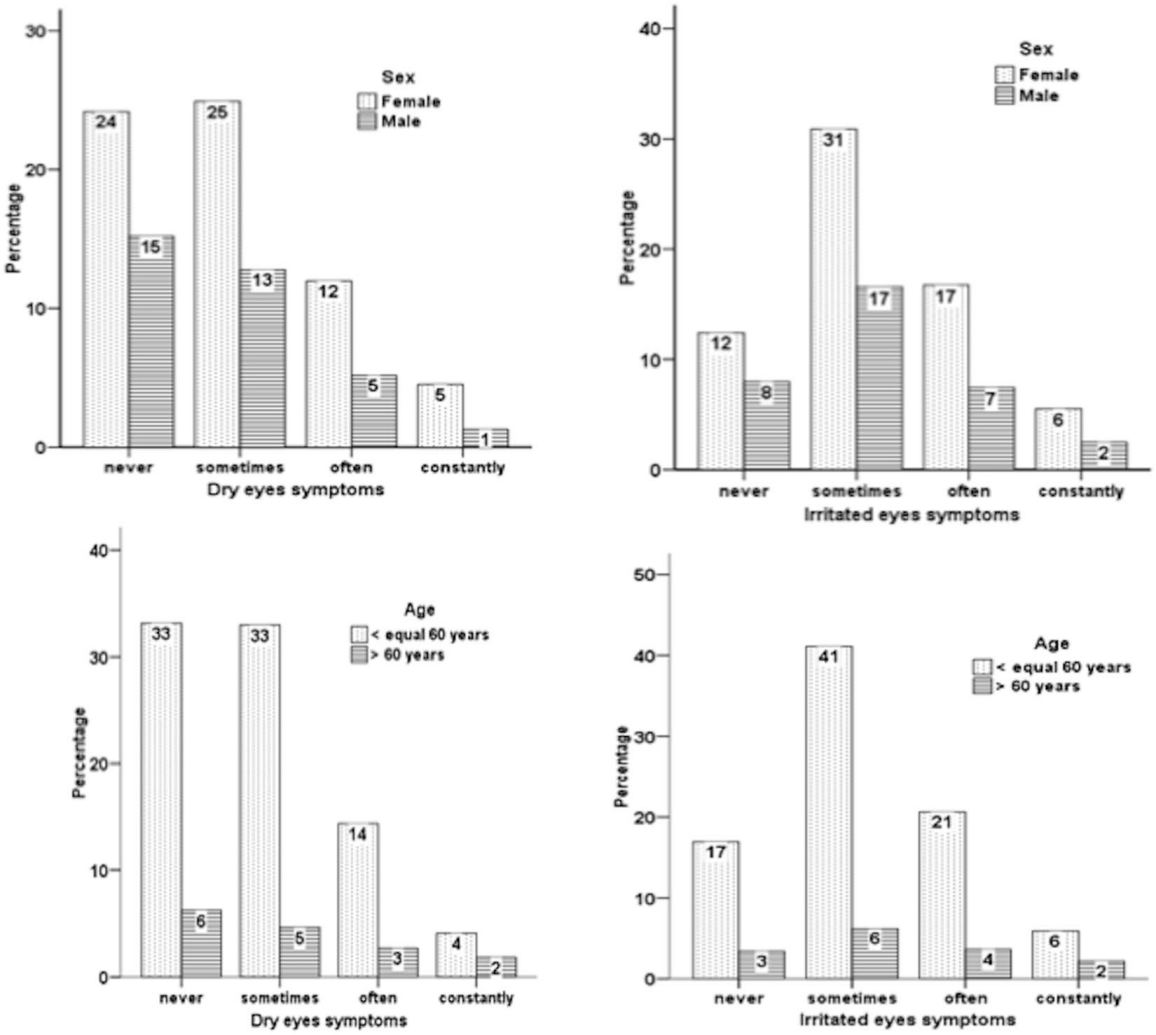
Frequency of dry eye symptoms according to sex and age. The dry eye symptoms of dryness and irritation were graded according to intensity (never, sometimes, often and constantly). Top left dryness according to sex, top right irritation according to sex; bottom left dryness according to age (<60 year-old/>60 years old) bottom right irritation according to age.

The overall prevalence of dry eye in this study population was 12.8% (398/3, 107), which represents the sum of individuals who reported a previous diagnosis of dry eye and individuals who reported severe symptoms of dry eye. In total, 10.2% (317/3, 107) participants reported a previous diagnosis of dry eye, and 4.9% (151/3, 107) reported severe symptoms, with 70 participants defined by both criteria. The distribution of dryness symptom was: 39.4% (1, 224) for never; 37.7% (1, 170) sometimes; 17.1% (531) often and 5.9% (182) constantly. Similarly, for irritation 20.3% (634) of participants assigned never; 47.5% (1, 475) sometimes; 24.2% (751) often and 8.0% (250) constantly. The prevalence of dry eye patients was 26.4% (105) for male and 73.6% (293) for females. Table 1 and Fig. 1 show the main characteristics of the study population by sex, age and frequency of risk factors, the distribution of symptoms as well as, isolated previous dry eye diagnosis and severe dry eye symptoms.

**Table 1:** Characteristics of the study population.

**Variable**	All n (%)	Dry eye group* n (%)	Previous dry eye diagnosis n (%)	Severe dry eye symptoms n (%)
Overall	3,107 (100)	398 (12.8)	317/3,107 (10.2)	151/3,107 (4.9)
**Sex**				
Male	1067 (34.4)	105 (26.4)	71 (28.7)	34 (22.5)
Female	2032 (65.6)	293 (73.6)	176 (71.3)	117 (77.5)
**Age category**				
18–39	1547 (50.3)	153 (38.9)	106 (43.5)	48 (32.2)
40–60	1052 (34.2)	139 (35.4)	86 (35.2)	52 (34.9)
60+	474 (15.3)	101 (25.7)	52 (21.3)	49 (32.9)
**Dryness symptom**				
Never	1224 (39.4)	12 (3.0)	12 (4.9)	—
Sometimes	1170 (37.7)	65 (16.3)	65 (26.3)	—
Often	531 (17.1)	161 (40.5)	161 (65.2)	—
Constantly	182 (5.9)	160 (40.2)	9 (3.6)	151 (100)
**Irritation symptom**				
Never	631 (20.3)	10 (2.5)	10 (4.0)	—
Sometimes	1475 (47.5)	99 (24.9)	98 (39.7)	—
Often	751 (24.2)	167 (42.0)	121 (49.0)	47 (31.1)
Constantly	250 (8.0)	122 (30.7)	18 (7.3)	104 (68.9)
**Frequency of risk factors**				
+60 years	474 (15.4)	101 (25.6)	52 (21.3)	49 (32.5)
≥40 years	1526 (49.5)	240 (60.8)	183 (58.3)	102 (67.5)
Diabetes	247 (7.9)	41 (10.3)	23 (9.3)	18 (11.9)
Menopause	294 (14.5)	70 (23.9)	35 (19.9)	35 (29.9)
Rheumatologic diseases	143 (4.6)	32 (8.0)	18 (7.3)	13 (8.6)
Cancer treatment	35 (1.1)	11 (2.8)	7 (2.8)	4 (2.6)
Smoking	193 (7.8)	30 (9.3)	14 (6.9)	16 (13.4)
Computer use >6 h per day	789 (25.4)	124 (31.2)	82 (33.2)	43 (28.5)
Ocular surgery	328 (10.6)	79 (19.8)	38 (15.4)	41 (27.2)
Contact lens wear	310 (10.0)	60 (15.1)	45 (18.2)	15 (9.9)
Antidepressants	257 (8.8)	62 (15.6)	38 (15.4)	24 (15.9)
Anti-Allergy medications	348 (11.2)	83 (20.9)	47 (19.0)	36 (23.8)

^+^Dry eye was defined as clinical previous dry eye diagnosis or the presence of severe dry eye symptoms.

The risk factors associated with dry eye in the univariate and multivariate analyses are displayed in Table 2 showing the logistic regression in the Brazilian study population in association with both, previous dry eye diagnosis and severe symptoms. The independent risk factors associated to dry eye in the univariate logistic regression analyses were female sex, 60 years and older, menopause, connective tissue disease, cancer treatment, contact lens wear, ocular surgery, anti-allergy and antidepressant medication, computer use >6 hours/day. Indeed, the subgroup of participants aged below 40 years old had an estimated protective risk of dry eye with OR 0.59 (95% CI: 0.47–0.73) *P* < 0.0001, for previous diagnosis and severe symptoms. The final multivariate model confirmed statistical significance such as female sex (OR 1.74; 95% CI: 1.12–1.93), age ≥60 year-old (OR 2.00; 95%CI: 1.44–2.77), history of ocular surgery (OR 1.84; 95%CI: 1.30–2.60), contact lens wear (OR 1.93; 95%CI: 1.36–2.73), cancer treatment (OR 3.03; 95%CI: 1.36–6.59), computer use >6 hours per day (OR 1.77; 95%CI: 1.36–2.31), antidepressants (OR 1.61; 95%CI: 1.12–2.31) and anti-allergy (OR 2.11; 95%CI: 1.54–2.89) medications. Regarding sex differences, a final multivariate model revealed that age >60 years, cancer treatment and anti-allergy medications twofold increased risk for dry eye in females, while antidepressant use importantly increased risk in male followed by ocular surgery, contact lens wear and anti-allergy medications.

**Table 2:** Risk factors for Dry Eye in Brazil.

Factors	Diagnosed dry eye [n = 317]			Severe symptoms [n = 151]			Previous dry eye + Severe symptoms [n = 398]		
	OR	95% CI	*P*	OR	95% CI	*P*	OR	95% CI	*P*
**Univariate analysis**									
Female sex	1.49	1.15–1.92	0.003	1.85	1.25–2.74	0.002	1.54	1.22–1.95	<0.0001
Age ≥40 years	1.47	1.16–1.87	0.001	2.20	1.55–3.12	<0.0001	1.68	1.36–2.09	<0.0001
Age ≥60 years	2.02	1.53–2.66	<0.0001	2.83	1.98–4.04	<0.0001	2.13	1.65–2.73	<0.0001
Diabetes	1.45	0.99–2.13	0.054	1.61	0.96–2.68	0.067	1.39	0.98–1.98	0.064
Connective tissue dis.	1.93	1.23–3.02	0.004	2.24	1.25–3.99	0.006	2.04	1.36–3.07	0.001
Ocular surgery	2.31	1.70–3.13	<0.0001	3.46	2.37–5.06	<0.0001	2.44	1.85–3.23	<0.0001
Cancer treatment	3.59	1.71–7.55	0.001	2.56	0.89–7.37	0.080	3.18	1.54–6.54	0.002
Contact lens wear	1.82	1.31–2.53	<0.0001	1.00	0.58–1.73	0.98	1.74	1.29–2.36	<0.0001
Anti-allergy drug	2.33	1.73–3.14	<0.0001	2.65	1.79–3.92	<0.0001	2.43	1.85–3.19	<0.0001
Computer use >6 h	1.49	1.16–1.92	0.002	1.14	0.79–1.64	0.48	1.39	1.10–1.75	0.005
Antidepressants	2.21	1.57–3.09	<0.0001	2.21	1.40–3.48	0.001	2.38	1.75–3.23	<0.0001
Smoking	1.44	0.83–2.48	0.18	1.91	1.10–3.31	0.020	1.25	0.83–1.88	0.281
Menopause	1.92	1.37–2.68	<0.0001	2.73	1.80–4.14	<0.0001	2.12	1.56–2.87	<0.0001
**Multivariate analysis**									
Female sex	1.47	1.12–1.93	0.005	1.91	1.28–2.84	0.001	1.53	1.20–1.96	0.001
Age ≥60 years	2.00	1.44–2.77	<0.0001	2.22	1.47–3.33	<0.0001	2.06	1.53–2.78	<0.0001
Ocular surgery	1.84	1.30–2.60	0.001	2.57	1.66–3.95	<0.0001	1.90	1.38–2.62	<0.0001
Cancer treatment	3.03	1.39–6.59	0.005	—	—	—	2.57	1.20–5.48	0.014
Contact lens wear	1.93	1.36–2.73	<0.0001	—	—	—	1.86	1.35–2.56	<0.0001
Anti-allergy drug	2.11	1.54–2.89	<0.0001	2.77	1.85–4.14	<0.0001	2.23	1.67–2.97	<0.0001
Computer use >6 h	1.77	1.36–2.31	<0.0001	—	—	—	1.65	1.29–2.11	<0.0001
Antidepressants	1.61	1.12–2.31	0.009	—	—	—	1.73	1.24–2.40	0.001
**Multivariate analysis**–**Female**									
Age ≥60 years	2.35	1.60–3.45	<0.0001	2.15	1.34–3.44	0.001	2.41	1.71–4.42	<0.0001
Ocular surgery	1.62	1.07–2.46	0.023	2.81	1.72–5.60	<0.0001	1.88	1.29–2.75	0.001
Cancer treatment	2.83	1.13–7.10	0.027	—	—	—	—	—	—
Contact lens wear	1.82	1.22–2.71	0.003	—	—	—	1.81	1.25–2.62	0.002
Anti-allergy drug	2.00	1.40–2.87	<0.0001	2.57	1.63–4.07	<0.0001	2.04	1.46–2.85	<0.0001
Computer use >6 h	1.93	1.42–2.63	<0.0001	—	—	—	1.78	1.34–2.37	<0.0001
Antidepressants	—	—	—	—	—	—	1.53	1.04–2.24	0.001
**Multivariate analysis**–**Male**									
Age ≥60 years	—	—	—	3.13	1.52–6.41	0.002	—	—	—
Ocular surgery	2.76	1.57–4.85	<00.0001	—	—	—	2.19	1.28–3.75	0.004
Cancer treatment	4.97	1.16–21.2	0.031	—	—	—	6.33	1.65–24.2	0.007
Contact lens wear	2.65	1.37–5.15	0.004	—	—	—	2.06	1.09–3.89	0.024
Anti-allergy drug	2.83	1.54–5.20	0.0.001	3.62	1.57–8.34	0.002	3.02	1.73–5.25	<0.0001
Computer use >6 h	—	—	—	—	—	—	—	—	—
Antidepressants	3.93	2.01–7.68	<0.0001	—	—	—	2.96	1.54–5.68	0.001

*Age ≥40 years - when inserted in the multivariate model was excluded due to higher impact of age ≥60 years.

**Figure 2:**
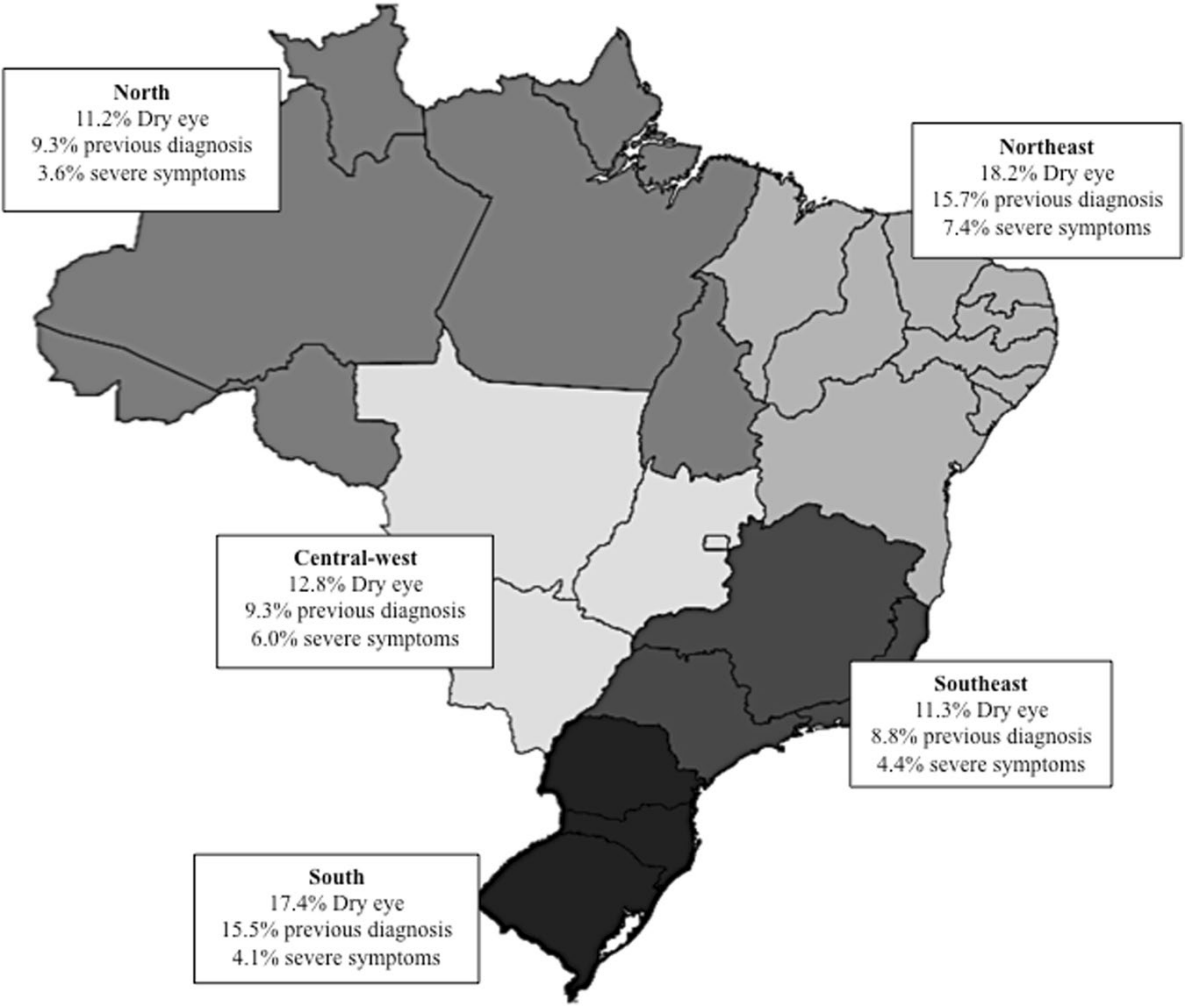
Prevalence of dry eye according to main geopolitical regions of Brazil (North, Northeast, Southeast, Central-west, South) as dry eye overall prevalence, severe symptoms and previous diagnosis. Map source http://www.clipartsfree.net/clipart/5496-colored-map-of-brazil-clipart.html; adapted by the authors.

The distribution by region of the questionnaires was 10.7% North; 9% Northeast; 17.7% Central-west; 53.3% Southeast and 9.3% South, somehow reflecting population demographic density trends around the country. Main characteristics and prevalence of dry eye by region are displayed in Table 3e and a Brazilian map Fig. 2. The Northeast region presented 18.2% dry eye diagnosis, followed by South 17.4%, Central-west 12.8%, Southeast 11.3% and North 11.2%, respectively. Table 4 shows variations of potential risk factors according to each one of the 5 regions in Brazil, using the bootstrap method for confirming results. By using this tool, after obtaining the final model stratified by Brazilian regions, the bootstrap model was applied to the analytic sample. This process was repeated 5,000 times to result in the model optimism that is the average across all the bootstrap iterations, and as a final step, it was obtained the optimism estimate from the apparent performance to get the optimism-corrected performance estimate. It means that when p-value of OR is similar with p-value of bootstrap, and then the original model can be obtained as well in the larger samples. As Table 4 presented, most results were confirmed, but North region did not have any variable able to obtain the similar p-value, so this region should have other variables influencing the dry eye and needs to have more specific studies, as the South region, the variable: “antidepressants use” did not confirm its influence in the large sample.

**Table 3:** Brazilian regions: climate and demographic characteristics and dry eye prevalence.

Region	North 331 (10.7)	Northeast 281 (9.0)	Central-west 549 (17.7)	Southeast 1658 (53.3)	South 288 (9.3)
Average annual temperature*	24–26 °C	20–28 °C	20–22 °C	20–24 °C	14–22 °C
Climate*	Equatorial	Tropical/semi-arid	Tropical	Tropical	Subtropical
Average annual rainfall (mm/year)*	3000	1000	1500-200	1500–2000	1250–2000
Demographic distribution* (total 203,191 million inhabitants)	17,285 (8.5%)	56,270 (27.7%)	15,268 (7.5%)	85,291 (42%)	29,077 (14.,3%)
Female	207 (62.5)	177 (63)	360 (66.5)	1131 (68.2)	157 (54.5)
Male	124 (37.5)	104 (37)	181 (33.5)	527 (31.8)	131 (45.5)
+60 years	39 (11.8)	65 (23.1)	147 (26.7)	189 (11.4)	34 (11.8)
Dry eye	37 (11.2)	53 (18.9)	70 (12.8)	188 (11.3)	50 (17.4)
Dry eye previous diagnosis	31 (9.3)	44 (15.7)	51 (9.3)	146 (8.8)	45 (15.6)
Dry eye severe symptoms	12 (3.6)	21 (7.4)	33 (6.0)	73 (4.4)	12 (4.1)

*Source: www.ibge.gov.br.

**Table 4:** Association between dry eye and potential risk factors by Brazilian regions.

Variable	Prevalence (%)	OR	CI 95%	p-value	*P*_bootstrap_*
**North (n = 331)**					n = 5,000
Anti-allergy use	34 (10.3)	2.59	1.05–6.36	0.038	**NS**
Contact lens wear	17 (5.1)	3.23	1.04–10.0	0.042	**NS**
**Northeast (n = 281)**					n = 5,000
Female sex	177 (63)	3.15	1.42–6.99	0.005	0.004
Contact lens wear	28 (9.9)	3.42	1.38–8.47	0.008	0.008
Anti-allergy use	32 (11.4)	3.01	1.28–7.07	0.012	0.011
Computer use	77 (27.4)	3.24	1.68–6.27	<0.0001	0.001
**Central-West (n** = **546)**					n = 5,000
Age >60 years	147 (26.9)	1.98	1.11–3.52	0.020	0.021
Anti-allergy use	28 (5.1)	4.15	1.77–9.73	0.001	0.001
Previous ocular surgery	106 (19.4)	2.40	1.31–4.39	0.004	0.005
**Southeast (n = 1642)**					n = 5,000
Female sex	1120 (68.2)	1.86	1.27–2.72	0.001	0.001
Age >60 years	189 (11.5)	2.75	1.77–4.27	<0.0001	<0.0001
Previous ocular surgery	136 (8.2)	2.97	1.88–4.68	<0.0001	<0.0001
Contact lens wear	197 (11.9)	1.92	1.25–2.96	0.003	0.004
Anti-allergy use	220 (13.4)	2.08	1.40–3.09	<0.0001	<0.0001
Computer use	427 (26.0)	1.69	1.18–2.40	0.004	0.003
**South (n** = **288)**					n = 5,000
Anti-allergy use	33 (11.4)	2.66	1.18–5.99	0.018	0.010
Antidepressants use	22 (7.6)	2.89	1.12–7.45	0.028	**NS**

*Bootstrapping method for confirming results based on 5,000 samples; CI = confidence interval; n = number; NS = non significant; OR = odds ratio; statistical significance *P* < 0.05.

## Discussion

This represents the first study of dry eye, comprising a large population sample in Latin America. It estimated the prevalence rates of dry eye according to previous diagnosis or presence of severe symptoms of dryness and irritation as well as risk factors impact, similarly to previous studies^9,16–18,22,23,29^. Some relevant particularities among the main geographic regions of the country, have regard to climate, environmental exposures and cultural conditions may probably contribute to the differences found in dry eye collected data. For instance, higher prevalence of dry eye was noted in Northeast region where prevails a semi-arid climate in contrast to the North region, which has humid equatorial features that could be protective.

Despite a broad range of dry eye numbers, the prevalence of dry eye found in this study resembles some other epidemiological studies performed around the world^7,10,18,22,29,33–35^. Several studies have reported rates of dry eye based on the report of severe symptoms and/or a physician’s diagnosis using the same questionnaire tool used herein. In those studies, the prevalence of disease ranged between 6.8 and 24.4% when considering previous diagnosis and 1.3 to 10.4% using severe symptoms, with higher numbers in Asian countries. Women consistently had a higher prevalence than men in all studies stratified by sex^9,17,18,23,29,30^. Our data also indicates a high frequency of less severe symptoms suggesting the milder cases of dry eye may be considerably high and negatively impact on dry eye patients’ visual acuity. This represents an important issue to be addressed by clinicians and eye care workers when dealing patients with eye discomfort and/or those reporting the most important related risk factors.

Increased age, female sex, menopause, visual display use over 6 hours per day, ocular history of surgery and contact lens wear, systemic conditions such as cancer treatment and connective tissue disorders as well as some medications have been extensively studied and were confirmed in this population sample with dry eye, although varying according to sex, age and geographical region. We found around two-fold increased risk for dry eye in participants over 60 years-old, anti-allergy medications, contact lens wear and ocular surgery history and visual display use more than 6 hours per day, while cancer treatment had three times increase. Interestingly, history of cancer treatment strongly correlated with dry eye in both females and males, age over 60 years doubled risk in women and not reaching significance in men, whereas the opposite was found related to antidepressants.

According to the official agency of statistics in Brazil (IBGE –Instituto Brasileiro de Geografia e Estatistica - www.ibge.gov.br), the overall proportion of female and males in the Brazilian population is around 55% women and 45% of men (female:male ratio of approximately 1.2:1), a difference that is higher in the group of our study population (female:male ratio of approximately 1.9:1). We believe that the higher proportion of women in our sample could be explained by two factors that were not anticipated when the recruitment strategy was designed. First, although we did not request that our collaborators recruited more women than men, we did not specifically stated that a 1:1 female:male ratio should be observed, as they enrolled participants consecutively. So, it is possible that women were overrepresented in the 4,000 questionnaires that were initially sent. Second, although we do not have empirical data about this, we believe that it is fair to assume that women are more receptive to answering these questionnaires than men, which could also have contributed to this difference. Therefore, if present, this selection bias was unintentional, and at random. Since dry eye is more common in women, we believe that this random bias does not jeopardize our conclusions. Besides that, to minimize this possibility it was also done an adjusted analysis by sex, as you can check in the multivariate analysis tables.

Some potential limitations of this study must be pointed out. First, in our study dry eye was diagnosed by the self-report of severe symptoms rather than a clinical evaluation of dry eye signs or a combination of both. Although dry eye has been recognized as a common ocular problem, its diagnosis remains a challenge, due to the lack of gold-standard methods and poor correlations among most commonly used tests^36,37^. Population-based studies evaluating dry eye differ somewhat in the definition of dry eye, in particular on whether using symptoms self-report and/or objective tests. It is important to acknowledge that, although, in some patients dry eye can only be diagnosed by a clinical set of tests^38^, there is an appreciation that since dry eye is mainly a symptomatic condition, the use of self-report questionnaires is a valuable tool for assessing the prevalence of dry eye in population-based studies^9,17,18^. Another limitation is the use of the self-report of a previous dry eye diagnosis for the purpose of our study. Although it allows the inclusion of patients in which dry eye was objectively diagnosed by clinical evaluation, it was beyond the purposes of this study to verify specificity and standardization of methods used. Therefore, we believe that this is a valid and complimentary strategy to include dry eye patients, which has also been used in other epidemiological studies. The design of our study aimed to include a large number of participants from different parts of the country, relying on the collaboration of individual ophthalmologists, so the option for a more comprehensive survey rather than including detailed demographic data, such as schooling, work activities and environmental exposure. Indeed, some documented risk factors of dry eye such as meibomian gland dysfunction, thyroid disease, hormone replacement, nutritional issues, and many others medications such as oral contraceptive, isotretinoin and anxiolytics were not investigated in this study. Despites these limitations, we truly believe that this study brings consistent information to fill gaps of dry eye data in Brazil and to inspire future studies designed to fulfill clinical information about dry eye, associated risks factors and impact of the disease on patients’ quality of life. We tried to achieve a broad sample and good representativeness of Brazilian population, once the large country size and demographic disparities imposed a great challenge for the study design.

In conclusion, our dry eye study in Brazil has been valuable in first describing dry eye prevalence in a large population sample from all five geopolitical regions of Brazil as well as in identifying some relevant risk factors. It was performed after a proper translation and validation of a short dry eye symptom questionnaire, which provides a useful tool for future studies.

## Methods

### Study population

This is a population cross-sectional study in all the 5 different geopolitical regions of Brazil. The study was carried out with the approval of the University of Campinas Institutional Research Ethics Committee Board and was conducted in accordance with the tenets of the Declaration of Helsinki and current legislation on clinical research. Written informed consent was obtained from all subjects after explanation of the procedures and study requirements.

The questionnaire used in this study was developed to evaluate dry eye disease prevalence in the Women Heath Study (WHS) performed in USA^9^. Thereafter, it is has been replicated in several population-based studies^17,18,22,23,29,30,39^. It comprises a short and simple tool, that consists of 3 items about previous diagnosis of dry eye from clinician; a range of dryness and irritation symptoms, as described below. An individual is considered positive for dry eye with reported rates of disease based on symptoms of dryness and irritation at least often and/or a physician’s diagnosis of dry eye, as reported by the participant. This questionnaire has been reported to have similar sensitivity and specificity as a 16 item instrument comprising symptom and also has been validated against a standardized clinical exam^31^. A translated and validated version of this short questionnaire was previously prepared^32^, to than, be distributed and applied as a cross-sectional survey in urban areas of all five different geopolitical regions of Brazil. Ophthalmologists who were former residents at University of Campinas who have been currently working on urban areas of one of these five geopolitical regions were contacted and invited to participate in this study. They were instructed to apply informed consents and questionnaires to participants aged 18 or older and selected from general population. In order to obtain a sample that was more representative of the population and avoid, as much as possible, selection bias, the following measures were applied: (i) recruiters were instructed to enroll participants from any labor activities, workplace environment, and socioeconomic status, consecutively; (ii) hospitalized patients and ophthalmological patients were not included. The latter aimed to avoid the overrepresentation of subjects with ophthalmic conditions in our sample, which could have occurred if recruitment targeted patients assisted by these collaborators in their clinics and hospitals. 4,000 questionnaires by mail, after contacting and receiving agreement from all coworkers listed in the acknowledgment footnote. Our study only includes urban areas, but they encompass 23 towns/cities from the 5 geopolitical regions of the county, with populations varying from 11,208 to more than 11,253,503 inhabitants.

### Diagnosis of dry eye

The validated short dry eye symptom questionnaire was applied to assess dry eye diagnosis and symptoms. In our study, dry eye was defined by self-report of a previous clinical diagnosis of dry eye or the presence of severe symptoms according to the questionnaire answers as described hereafter. Dry eye diagnosis was established by a self-report of previous diagnosis of dry eye by the question: “Have you ever been diagnosed (by a clinician) as having dry eye disease?” yes or no answer. Whereas, dry eye symptoms were evaluated in the following questions: “How often do your eyes feel dry? And “How often do your eyes feel irritated?” which should have been answered as “never”, “sometimes”, often” and “constantly”. Thus, dry eye was defined as (1) affirmative answer to previous dry eye diagnosis or (2) the presence of severe symptoms (both dryness and irritation indicated as constantly or often). Indeed, we evaluated separately dry eye diagnosis and severe symptoms. As mentioned, this questionnaire was previously used in other population studies and was reported to provide a high specificity for dry eye^31,17^.

### Risk factors for dry eye

Demographic data and a list of conditions possibly associated with dry eye were included in the questionnaire to evaluate the impact of potential risk factors for this condition. These included: age, sex, menopause, diabetes, connective tissue disorders, cancer therapy, history of ocular surgery, computer use more than 6 hours per day, smoking (current smoker self-report status), contact lens wear and medications (antidepressants and anti-allergy).

### Statistical analysis

As a cross-sectional study demands it was estimated the prevalence and risk factors of dry eye.We calculated the prevalence of dry eye in the overall study population, as well as according to sex, age categories and Brazilian geopolitical regions (North, Northeast, Central-west, Southeast and South). To identify risk factors associated with dry eye the OR (odds ratio) and 95% Confidence Intervals (CI) were calculated using the univariate logistic regression. After that, it was applied the multivariate logistic regression analysis isolated and after that adjusted by region, considering statistically significant a lower *P* value of 0.05. To check the stability of final models and validate the confidence intervals, it was applied the bootstrap test, through random repeated samples, mainly when stratified analyses by five Brazilian regions were done. Due to wide used of the bootstrap for correcting the inherent optimism in estimates of model performance obtained on the same sample, it was applied in our study^40^. The optimism is defined as the difference between the bootstrap performance and its out-of-sample performance. First, a prediction model was developed in the analytic sample (the original sample). Then, the apparent performance of the estimated model in the analytic sample was accessed, to be applied in a bootstrap sample with replacement from the original analysis sample and developing a prediction model in this bootstrap sample that ends with a stable final model with its confidence intervals. All cautions were given to perform each step, such as selection of predictors and estimation of regression coefficients. All statistical analyses were applied using SPSS 21.0 software (SPSS Incorporation, Chicago, IL, USA).

## Electronic supplementary material

Supplementary file
