# Supplementary material for: Prevalence and Risk Factors of self-reported dry eye in Brazil using a short symptom questionnaire
Source: Sci Rep. 2018 Feb 1;8:2076. doi: 10.1038/s41598-018-20273-9 (PMC5794758; doi:10.1038/s41598-018-20273-9)
Supplement: Supplementary file 1 — Supplementary file [file 41598_2018_20273_MOESM1_ESM.pdf]

Title page

Prevalence and Risk Factors of self-reported dry eye in Brazil using a short symptom questionnaire

Authors

Julia Silvestre de Castro, MS, Iara Borin Selegatto MS, Rosane Silvestre de Castro MD, PhD, Eliana C. M. Miranda M.Ed. PhD, José Paulo Cabral de Vasconcelos MD, PhD, Keila Monteiro de Carvalho MD, PhD, Carlos Eduardo Leite Arieta MD, PhD, Monica Alves MD, PhD

University of Campinas – UNICAMP

Dry eye questionnaire:

Age:___________________

Sex: ( ) male ( ) female

1.How often do your eyes feel dry?

( ) never

( ) sometimes

( ) often

( ) constantly

2.How often do your eyes feel irritated?

( ) never

( ) sometimes

( ) often

( ) constantly

3.Have you ever been diagnosed (by a clinician) as having dry eye syndrome?

( ) Yes ( ) No

Mark the any of the following conditions if you have:

Menopause ( ) Yes ( ) No

Diabetes ( ) Yes ( ) No

Connective tissue disorders ( ) Yes ( ) No

Cancer therapy ( ) Yes ( ) No

Ocular surgery ( ) Yes ( ) No

Contact lens wear ( ) Yes ( ) No

Computer use more than 6 hours per day ( ) Yes ( ) No

Smoking ( ) Yes ( ) No

Antidepressants medications ( ) Yes ( ) No

Anti-allergy medications ( ) Yes ( ) No
